# Biological characterization of a clinical human adenovirus type 3 isolate with oncolytic potential

**DOI:** 10.3389/fmicb.2026.1912909

**Published:** 2026-09-23

**Authors:** Zhiwei Shen, Rui Tian, Tianhao Wu, Xiaojing Ou, Jiaqi Zhang, Zhiyuan Li, Meiyuan Gong, Meng Meng, Luyao Ji, Zhenyu Rong, Yaxuan Sun, Xuyue Zhang, Zeyang Tang, Yue Li, Wenqing Yu, Feng Han, Yonglin Yang, Wenlong Dai

**Affiliations:** 1 Department of Pharmaceutics, National Vaccine Innovation Platform, School of Pharmacy, Nanjing Medical University, Nanjing, China; 2 Nanjing Medical University Taizhou Institute of Pharmaceutical Industry, Taizhou, China; 3 Medical Basic Research Innovation Center for Cardiovascular and Cerebrovascular Diseases, Ministry of Education, Brain Health Innovation Center, School of Pharmacy, Nanjing Medical University, Nanjing, China; 4 Department of Infectious Diseases, Institute of Hepatology, Taizhou School of Clinical Medicine Nanjing Medical University, The Affiliated Taizhou People's Hospital of Nanjing Medical University, Taizhou, China; 5 Jiangsu Province Innovation Center for Brain-Inspired Intelligence Technology, Nanjing, China; 6 Research Center for Pharmaceutical Synthetic Biology, School of Pharmacy, Nanjing Medical University, Nanjing, China; 7 Key Laboratory of Immunoregulation and Intervention for Major Chronic Diseases of Jiangsu Province, Nanjing, China; 8 NHC Key Laboratory of Antibody Technique, Nanjing Medical University, Nanjing, China

**Keywords:** apoptosis, clinical isolate, human adenovirus type 3, oncolytic virotherapy, viral tropism

## Abstract

**Introduction:**

Human adenovirus type 3 (HAdV-3) is a major adenovirus type associated with respiratory infections in China. Despite its clinical importance, the biological characteristics and potential biomedical applications of contemporary circulating HAdV-3 strains remain incompletely understood. Naturally occurring species B adenoviruses also remain relatively underexplored as potential starting backbones for oncolytic virotherapy. This study aimed to characterize a contemporary clinical HAdV-3 isolate and evaluate its viral genome amplification, apoptosis induction, and antitumor activity.

**Methods:**

A clinical HAdV-3 isolate, designated HAdV-3-TZ01, was recovered from a throat swab specimen from a patient with adenovirus-associated respiratory disease. Viral identity and genomic characteristics were determined using multiplex PCR, Sanger sequencing, whole-genome sequencing, phylogenetic analysis, and recombination analysis. Viral genome amplification kinetics were evaluated in human respiratory-related and tumor-derived cell lines and a murine cell line by quantitative PCR. Comparative apoptosis induction by HAdV-3-TZ01 and HAdV-5 was assessed in 293F cells infected at a low multiplicity of infection using Annexin V/PI flow cytometry. Antitumor activity was evaluated following repeated intratumoral administration of HAdV-3-TZ01 in an A549-luciferase xenograft model in BALB/c-nu nude mice, with tumor growth and bioluminescence monitored longitudinally.

**Results:**

Genomic and phylogenetic analyses confirmed that HAdV-3-TZ01 belonged to HAdV-3 within species B and exhibited a genomic backbone highly conserved with contemporary circulating HAdV-3 strains. A short putative HAdV-7-derived recombinant fragment was identified. HAdV-3-TZ01 showed efficient viral genome amplification in multiple human respiratory-related cell lines, including A549, H3122, BEAS-2B, MRC-5, and HEK293FT cells, whereas genome amplification was markedly restricted in murine L929 cells. HAdV-3-TZ01 induced a significantly higher proportion of late apoptotic cells than HAdV-5 in 293F cells under low-multiplicity infection conditions, although the absolute increase in total apoptosis was modest. In the A549-luciferase xenograft model, intratumoral administration of HAdV-3-TZ01 significantly suppressed tumor progression. By day 43, mean tumor volume was reduced by approximately 54% and tumor-associated bioluminescence by approximately 50% compared with mock-treated controls. No significant body-weight loss or overt signs of distress were observed under the experimental conditions.

**Conclusion:**

HAdV-3-TZ01 retains the characteristic human epithelial tropism of HAdV-3 and exhibits efficient viral genome amplification in human epithelial-derived cells, apoptosis induction, and measurable antitumor activity in vivo. The apoptosis difference between HAdV-3-TZ01 and HAdV-5 should be interpreted cautiously because the analysis was limited to 293F cells and did not establish a tumor-specific pro-apoptotic mechanism. As an unmodified respiratory pathogen, HAdV-3-TZ01 should not be considered a directly translatable therapeutic agent. Rather, these findings support HAdV-3-TZ01 as a potential starting backbone for future genetic engineering aimed at improving tumor selectivity and safety.

## 1 Introduction

Human adenovirus type 3 (HAdV-3) is a major cause of adenovirus-associated respiratory disease in China and is frequently associated with outbreaks among children and young adults (Fang et al., 2025; Liu et al., 2023). In China, HAdV-3 has consistently been identified as a major contributor to adenovirus-associated respiratory diseases and is often linked to severe pneumonia and community-wide epidemics (Chen et al., 2022; Fang et al., 2025; Sun et al., 2022; Wen et al., 2021; Zhou et al., 2024). Despite extensive epidemiological surveillance, the biological characteristics of newly circulating HAdV-3 strains remain incompletely understood, particularly with respect to their replication properties and potential biomedical applications.

Beyond their role as important respiratory pathogens, adenoviruses have attracted considerable attention as versatile platforms for gene delivery, vaccine development, and cancer therapy (Sato-Dahlman et al., 2020; Wang et al., 2023). Their well-characterized genome, high transduction efficiency, large transgene carrying capacity, and established manufacturing processes have facilitated the development of numerous adenovirus-based therapeutics. In recent years, oncolytic adenoviruses have emerged as a promising class of anticancer agents capable of selectively replicating within tumor cells and inducing direct tumor lysis while simultaneously stimulating antitumor immune responses (Tazawa et al., 2017; van Beusechem et al., 2002). Several adenovirus-based oncolytic therapeutics have advanced into clinical development. For example, CG0070 (cretostimogene grenadenorepvec), an oncolytic adenovirus expressing GM-CSF, has demonstrated clinical activity in patients with non-muscle-invasive bladder cancer (Tang et al., 2026). In addition, various engineered adenoviral platforms expressing immunomodulatory transgenes are currently being investigated in preclinical and clinical studies (Grandi et al., 2023; Watanabe et al., 2021; Yang et al., 2022).

Historically, HAdV-5 has been the predominant backbone for the development of oncolytic adenoviruses (Gao et al., 2020; Qian et al., 2025). Although HAdV-5-based vectors have demonstrated promising therapeutic potential, their high seroprevalence of neutralizing antibodies and dependence on specific cellular receptors may limit their performance in certain clinical settings (Hong et al., 2025). For example, while HAdV-5 neutralizing antibodies are highly prevalent in the population, the seroprevalence of HAdV-3 neutralizing antibodies in children is relatively low (12.07–33.96%), suggesting that HAdV-3-based vectors may potentially encounter fewer pre-existing immunity constraints (Lu et al., 2023). In addition, species B adenoviruses such as HAdV-3 utilize alternative cellular receptors, including CD46 and desmoglein-2 (DSG2) (Gaggar et al., 2003; Hensen et al., 2020; Wang et al., 2011), which are broadly expressed in epithelial tissues and many human malignancies (Sharma et al., 2009). Nevertheless, the biological properties and therapeutic potential of naturally circulating HAdV-3 isolates remain insufficiently characterized.

In the present study, we isolated a contemporary clinical HAdV-3 strain, designated HAdV-3-TZ01, from a patient with acute respiratory infection. Using whole-genome sequencing and phylogenetic analysis, we characterized its genetic features and evaluated its viral genome amplification capacity in respiratory epithelial and tumor-derived cell lines. In addition, we compared the apoptotic responses between HAdV-3-TZ01 and HAdV-5 during infection spread and assessed its antitumor efficacy in an A549-luciferase xenograft mouse model. Our findings provide updated biological insights into a currently circulating HAdV-3 isolate and support the further exploration of HAdV-3 as a potential platform for oncolytic virotherapy.

## 2 Materials and methods

### 2.1 Cells and viruses

HEK293FT, A549, BEAS-2B, H3122, MRC-5, and L929 cell lines were maintained in our laboratory. Cells were cultured in Dulbecco’s Modified Eagle Medium (DMEM) (#BC-M-005, BioChannel, China) supplemented with 10% fetal bovine serum (FBS) (#BC-SE-FBS07, BioChannel, China) at 37 °C in a humidified incubator containing 5% CO₂. HEK293FT cells (an adherent derivative of HEK293 expressing the SV40 large T antigen) were used for virus propagation and TCID₅₀ titration. 293F cells (a suspension-adapted derivative of HEK293 maintained in serum-free medium) were used exclusively for apoptosis analysis. Both cell lines are derived from human embryonic kidney cells and are not tumor-derived. The suspension growth characteristics of 293F cells facilitate uniform cell preparation and flow cytometric analysis. HAdV-3-TZ01 and HAdV-5 used in the comparative apoptosis assay were propagated and titrated using the same HEK293FT cell system to ensure consistency of viral preparations. Wild-type human adenovirus type 5 (HAdV-5) was obtained from Guangzhou Paizhen Biotechnology Co., Ltd. (#WRS-240802, Guangzhou, China). The HAdV-3 isolate used in this study, designated HAdV-3-TZ01, was isolated from a clinical throat swab specimen and propagated in HEK293FT cells before characterization and subsequent experiments.

### 2.2 Clinical sample collection and virus isolation

A throat swab specimen was collected from a 5-year-old female patient admitted to The Affiliated Taizhou People’s Hospital of Nanjing Medical University, Taizhou, China, on May 23, 2024. The patient presented with fever and cough for 4 days before admission. Respiratory pathogen testing revealed adenovirus DNA positivity, and chest radiography indicated bronchopneumonia. The specimen was collected during hospitalization for adenovirus isolation and further characterization. Samples were transported on ice and processed within 12 h after collection. The specimen was diluted 1:1 in phosphate-buffered saline (PBS), clarified by centrifugation at 1,500 × *g* for 10 min, and filtered through a 0.22 μm membrane. The filtrate was supplemented with penicillin (100 IU/mL) and streptomycin (100 μg/mL) before inoculation.

HEK293FT cells were maintained in DMEM supplemented with 10% FBS at 37 °C in a humidified atmosphere containing 5% CO₂. For virus isolation, confluent HEK293FT cell monolayers were inoculated with clarified clinical specimens and incubated for 2 h to allow viral adsorption. After removal of the inoculum, fresh maintenance medium containing 2% FBS was added. Cells were monitored daily for cytopathic effects (CPE). Blind passaging was performed every 5–7 days in the absence of visible CPE. Virus-containing supernatants were harvested following the appearance of characteristic adenoviral CPE, clarified by low-speed centrifugation, aliquoted, and stored at −80 °C. Third-passage (P3) virus stocks were used for subsequent experiments.

For *in vivo* administration, the P3 virus stock was concentrated by sucrose cushion ultracentrifugation. Briefly, virus-containing supernatants together with infected cell pellets were subjected to three cycles of freeze–thaw (−80 °C/37 °C) to release intracellular viral particles, followed by low-speed centrifugation (3,000 × *g*, 4 °C, 20 min) to remove cellular debris. The clarified supernatant was layered onto a 20% sucrose cushion prepared in PBS (pH 7.4) and centrifuged at 100,000 × *g* for 2 h at 4 °C. The viral pellet was resuspended in sterile PBS and stored at −80 °C. The resulting concentrated virus stock was used for subsequent *in vivo* experiments. The viral titer was determined by TCID₅₀ assay as described below, yielding 3.16 × 10^11^ TCID₅₀/mL.

### 2.3 Transmission electron microscopy (TEM) analysis

Transmission electron microscopy (TEM) analysis was performed by Shanghai Weishi Biotechnology Co., Ltd. (Shanghai, China). Briefly, 5 μL of virus-enriched sample was placed onto a Formvar–carbon-coated copper grid and incubated for 5 min, followed by blotting with filter paper. The samples were fixed with 1% glutaraldehyde (final concentration) in 0.1 M phosphate buffer (pH 7.4) for 1 min. After washing with phosphate buffer, the grids were negatively stained with 1% uranyl acetate. Images were acquired using a transmission electron microscope (Talos 120, FEI, USA) according to standard operating procedures.

### 2.4 Virus titration by TCID₅₀ assay

Virus infectivity was determined by a tissue culture infectious dose 50% (TCID₅₀) assay in 293FT cells. Briefly, 293FT cells were seeded into 96-well plates at 1 × 10^4^ cells per well and cultured overnight. Virus stocks were serially diluted 10-fold in DMEM containing 2% FBS, and 100 μL of each dilution was added to eight replicate wells. After incubation at 37 °C in a humidified 5% CO₂ atmosphere, the wells were monitored daily for the appearance of cytopathic effects (CPE). CPE-positive and CPE-negative wells were recorded at 3 days post-infection, and viral titers were calculated using the Reed–Muench method and expressed as TCID₅₀/mL.

### 2.5 Viral identification and whole-genome sequencing

Viral DNA was extracted from infected-cell culture supernatants using a Universal Genomic DNA Kit (#CW2298M, Cowin Biotech Co., Ltd., China) according to the manufacturer’s instructions. Preliminary species identification was performed using a previously described multiplex PCR assay targeting conserved regions of the adenoviral hexon gene (Xu et al., 2000). PCR products were purified and subjected to Sanger sequencing. Consensus sequences were compared against the NCBI nucleotide database using BLAST for preliminary virus typing.

For whole-genome characterization, viral genomic DNA was submitted to a commercial sequencing service provider for next-generation sequencing. Sequencing libraries were prepared according to standard protocols and sequenced using paired-end 150-bp reads on an Illumina NovaSeq platform. Raw sequencing reads were subjected to quality control and *de novo* assembly to obtain the complete viral genome sequence. Open reading frames were annotated based on sequence homology with representative adenovirus reference genomes available in GenBank.

### 2.6 Phylogenetic and recombination analyses

Representative human adenovirus reference sequences were retrieved from GenBank. Multiple sequence alignments were generated using MEGA version 11 (Tamura et al., 2021). The optimal nucleotide substitution model was selected using the “Find Best DNA/Protein Models” function implemented in MEGA. Phylogenetic trees were constructed using the maximum-likelihood method with 1,000 bootstrap replicates.

Potential recombination events were identified using RDP4 (v4.101) with multiple detection algorithms under default settings (Martin et al., 2015). Putative recombination signals were further evaluated using SimPlot (v3.5.1) based on similarity plot and bootscanning analyses (Lole et al., 1999).

### 2.7 Comparative apoptosis analysis

293F cells (a suspension-adapted derivative of HEK293 maintained in serum-free medium) were used for this assay. This cell line was chosen because its suspension growth characteristics facilitate uniform cell preparation and high-throughput flow cytometric analysis, which is technically advantageous for detecting subtle differences in apoptotic populations. Cells were cultured in Trans CD05 medium (#YYCD05, Youyi Biotechnology, China) at 37 °C with 5% CO₂ in a shaking incubator (125 rpm). Cells were infected with HAdV-3 or HAdV-5 at a multiplicity of infection (MOI) of 0.001. HAdV-3-TZ01 and HAdV-5 used in this comparative assay were propagated and titrated using the same HEK293FT cell system to ensure consistency of viral preparations. Following infection, cell samples were collected at 48 h post-infection for apoptosis analysis.

Apoptosis was assessed using an Annexin V-FITC/PI Apoptosis Detection Kit (#C1062M, Beyotime, China) according to the manufacturer’s instructions. Briefly, cells were harvested by centrifugation, washed once with cold PBS, and resuspended in Annexin V binding buffer. Annexin V-FITC and propidium iodide (PI) were added sequentially, and samples were incubated for 15 min at room temperature in the dark. Flow cytometric analysis was performed using a CaleCyter2080 flow cytometer (Calerio, China). At least 10,000 events were acquired for each sample.

Data were analyzed using FlowJo V10 software. Viable cells, early apoptotic cells (Annexin V^+^/PI^−^), and late apoptotic cells (Annexin V^+^/PI^+^) were quantified. Total apoptosis was defined as the sum of early and late apoptotic populations. All experiments were performed in three independent biological replicates.

### 2.8 Cell tropism and viral genome amplification

Human lung carcinoma A549 cells, human bronchial epithelial BEAS-2B cells, human lung adenocarcinoma H3122 cells, human lung fibroblast MRC-5 cells, human embryonic kidney 293FT cells, and murine fibroblast L929 cells were cultured in DMEM supplemented with 10% FBS.

Cells were seeded in 12-well plates at a density of 1 × 10^5^ cells per well and infected with HAdV-3-TZ01 at a multiplicity of infection (MOI) of 0.1, calculated based on TCID₅₀ titers. Following viral adsorption for 1–2 h, the inoculum was removed and replaced with fresh maintenance medium. Mock-infected cells received culture medium alone. Samples were collected at 12, 24, 48, 72, and 96 h post-infection for viral genome quantification. Total cellular DNA was extracted using a Universal Genomic DNA Kit according to the manufacturer’s instructions. Viral genome levels were quantified by quantitative PCR (qPCR) targeting the conserved adenoviral hexon gene region using previously described primers (Qiu et al., 2018). Primer sequences are listed in Table 1. The β-actin gene was used as an internal reference for normalization.

**Table 1:** Primers used for quantitative PCR analysis.

Target gene	Primer	Sequence (5′–3′)	Amplicon size (bp)	Reference
Adenovirus hexon	Hexon-F	AGACATGACHTTYGAGGTGGAYCC	122	Qiu et al. (2018)
Adenovirus hexon	Hexon-R	CGCAGGTASACBGYYTCRA	122	Qiu et al. (2018)
β-actin	Actin-F	GAGGCCCAGAGCAAGAGAG	107	This study
β-actin	Actin-R	TTGTAGAAGGTGTGGTGCCAG	107	This study

Primer sequences used for quantification of adenoviral genome copy numbers by qPCR. The adenoviral hexon gene was used for viral genome detection, and β-actin served as the internal reference gene. Amplicon sizes and literature sources are indicated.

qPCR reactions were performed using SYBR Green Master Mix (#11184ES08, Yeasen Biotechnology Co., Ltd.) on a Gentier 96R Real-Time PCR System (Xi’an Tianlong Science and Technology Co., Ltd.) according to the manufacturer’s instructions. Relative viral genome levels were calculated using the 2^−ΔΔCt^ method. Viral genome levels at different time points were normalized to the 12 hpi sample within each cell line, which was defined as the baseline level, and normalized values were transformed to log10 scale for visualization and statistical analysis. Three independent biological replicates were performed for each condition.

Statistical analyses were performed using GraphPad Prism. Differences among cell types and infection time points were analyzed using two-way ANOVA with interaction effects, followed by Šídák’s multiple comparisons test. A *p* value <0.05 was considered statistically significant.

### 2.9 Xenograft tumor model and *in vivo* evaluation

Sixteen female BALB/c-nu nude mice (4 weeks old, weighing approximately 18–20 g) were used in this study. Sample size (*n* = 8 per group) was determined empirically based on previous experience with this tumor model. Each mouse was subcutaneously inoculated in the right axillary region with 4 × 10^6^ A549-luciferase (A549-Luc) cells suspended in 100 μL PBS. Tumor growth was monitored every 3 days using digital calipers, and tumor volume was calculated using the formula *V* = *L* × *W*^2^/2, where *L* represents the longest diameter and *W* represents the shortest diameter.

When tumors reached approximately 100 mm^3^, mice were stratified according to tumor volume and randomly assigned to treatment and control groups (*n* = 8 per group) using a computer-generated randomization sequence. Mice in the treatment group received intratumoral injections of HAdV-3-TZ01 (50 μL per dose, containing 1.58 × 10^10^ TCID₅₀; stock titer: 3.16 × 10^11^ TCID₅₀/mL) on days 21 and 34 after tumor implantation. The two-injection schedule (days 21 and 34, with a 13-day interval) was selected based on previously reported intratumoral dosing strategies for oncolytic adenovirus therapies. Similar dosing intervals have been evaluated in clinical studies, such as the phase 1 BETA prime trial of AdAPT-001, in which intratumoral administration of the oncolytic adenovirus was performed once every 2 weeks at the recommended phase 2 dose (Conley et al., 2024). This is consistent with the principle that sustained, multi-dose regimens can achieve superior therapeutic outcomes compared with single or short-term dosing schedules. Control mice received equal volumes of PBS following the same schedule. Tumor volume measurements and bioluminescence imaging analyses were performed by investigators blinded to group allocation.

The viral dose was selected based on previously reported intratumoral administration regimens of oncolytic adenoviruses in murine xenograft models, taking into consideration the tumor size and feasibility of intratumoral administration. Preliminary tolerability observations showed no apparent body weight loss or treatment-related distress during the treatment period. Body weight was recorded throughout the study to evaluate treatment-associated toxicity. No formal dose-escalation or dose-optimization study was performed.

Mice were monitored daily for signs of distress or discomfort throughout the study. Humane endpoints were defined as tumor volume exceeding 1,500 mm^3^, tumor ulceration, >20% body weight loss, or signs of severe pain/distress. Animals reaching humane endpoints were euthanized by CO₂ inhalation followed by cervical dislocation. No animals were excluded from the analysis.

All animal experiments were conducted in accordance with institutional guidelines and were approved by the Institutional Animal Care and Use Committee (Approval No. 2410053).

### 2.10 Bioluminescence imaging

Tumor burden was monitored by bioluminescence imaging (BLI) on days 20, 22, 32, and 43 after tumor implantation. Mice received intraperitoneal injections of D-luciferin (#40901ES01, Yeasen, China) (15 mg/mL) at 10 μL/g body weight approximately 15 min before imaging. Images were acquired using an Aniview SE small-animal imaging system under isoflurane anesthesia. Bioluminescence signals were quantified as photon flux (photons/s/cm^2^/sr) using the manufacturer’s analysis software.

### 2.11 Statistical analysis

All statistical analyses were performed using GraphPad Prism 10. Data are presented as mean ± SEM. Comparisons among multiple groups in apoptosis assays were performed using one-way ANOVA followed by Tukey’s multiple-comparisons test. Tumor volume, bioluminescence intensity, and body weight data were analyzed using two-way repeated-measures ANOVA with Geisser–Greenhouse correction followed by multiple-comparisons tests. A *p* value < 0.05 was considered statistically significant.

## 3 Results

### 3.1 Isolation and genomic characterization of HAdV-3-TZ01

A clinical adenovirus isolate, designated HAdV-3-TZ01, was recovered from a throat swab sample collected from a patient presenting with mild respiratory symptoms. Following inoculation onto HEK293FT cells, typical adenoviral cytopathic effects (CPE), characterized by cell rounding, aggregation, and detachment, became evident within 3 days post-infection and were readily observed after two rounds of blind passage (Figures 1A,B), indicating successful viral isolation and propagation.

**Figure 1:**
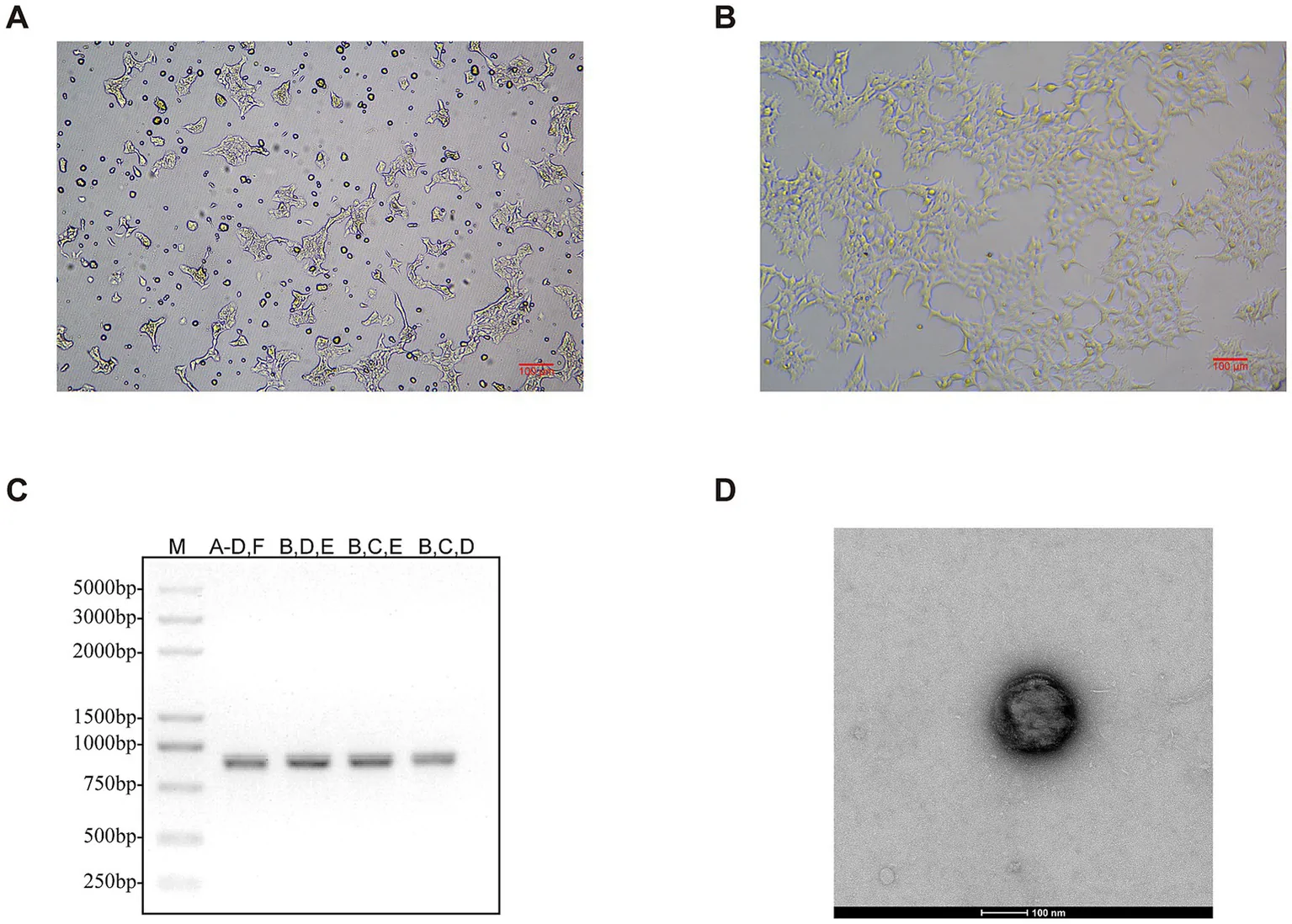
Isolation, identification, and morphological characterization of HAdV-3-TZ01. **(A)** Representative micrograph of HEK293FT cells showing typical cytopathic effects induced by HAdV-3-TZ01 during virus isolation and propagation. Representative images obtained during the third passage. Scale bar = 100 μm; magnification 100×; imaging conditions (bright-field microscopy). **(B)** Representative micrograph of mock-treated HEK293FT cells showing normal cell morphology. Scale bar = 100 μm; magnification 100×; imaging conditions (bright-field microscopy). **(C)** Agarose gel electrophoresis analysis of multiplex PCR products used for adenovirus typing. Viral supernatants collected from infected HEK293FT cells were used as templates for multiplex PCR amplification with previously reported primer sets. PCR products were separated on a 1% agarose gel and visualized using a gel imaging system. Lane M indicates the DNA molecular weight marker. **(D)** Representative transmission electron micrograph of HAdV-3-TZ01 particles concentrated by low-speed centrifugation (13,000 rpm). Adenovirus-like particles with characteristic morphology were observed (scale bar = 100 nm).

To determine the identity of the isolate, viral DNA was extracted from infected-cell supernatants and subjected to multiplex PCR using previously described adenovirus species-specific primer sets targeting conserved regions of the hexon gene. As shown in Figure 1C, amplification products of approximately 750–1,000 bp were detected with multiple primer combinations. The observed amplification profile was consistent with species B adenoviruses and differed from the characteristic patterns reported for other adenovirus species. Subsequent Sanger sequencing of the PCR products confirmed that the isolate belonged to human adenovirus species B and shared the highest sequence similarity with human adenovirus type 3 (HAdV-3).

Transmission electron microscopy (TEM) further revealed viral particles with typical adenoviral morphology (Figure 1D), providing additional structural confirmation of the isolate.

To further characterize the isolate, whole-genome sequencing was performed using high-throughput sequencing technology (Figure 2A). The complete viral genome was assembled and compared with representative human adenovirus reference strains. Phylogenetic analysis based on the full-length genome demonstrated that the isolate clustered robustly with HAdV-3 strains and was clearly separated from other adenovirus types (Figure 2B). Consistent results were obtained using analyses of major capsid genes, confirming the classification of the isolate as HAdV-3 (Figures 2C–E). Based on these findings, the virus was designated HAdV-3-TZ01.

**Figure 2:**
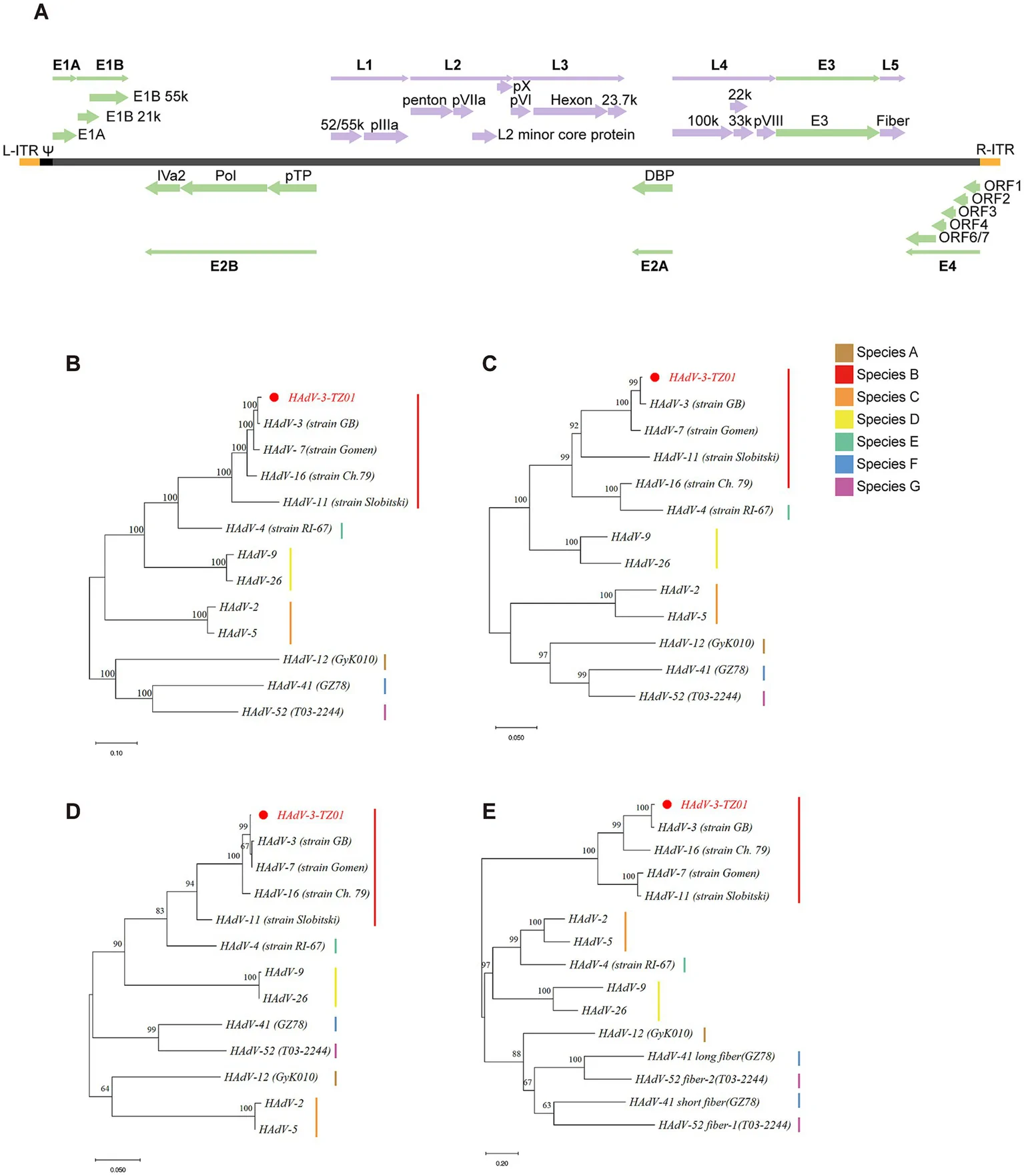
Genomic organization and phylogenetic characterization of HAdV-3-TZ01. **(A)** Schematic representation of the complete genome organization of HAdV-3-TZ01. Major early (E1A, E1B, E2, E3, and E4), intermediate (IX and IVa2), and late (L1–L5) transcription units, as well as the inverted terminal repeats (ITRs), are indicated according to their genomic locations and transcriptional orientations. Representative genes, including hexon, penton base, fiber, DNA polymerase, pre-terminal protein (pTP), DNA-binding protein (DBP), and 100 K, are labeled. **(B–E)** Maximum-likelihood phylogenetic analyses of HAdV-3-TZ01 and representative human adenoviruses based on the complete genome sequence **(B)**, hexon protein **(C)**, penton base protein **(D)**, and fiber protein **(E)**. The best-fit substitution models were selected using the model selection function implemented in MEGA11 and are indicated in the corresponding trees. Bootstrap values (1,000 replicates) are shown at the nodes, and scale bars represent the number of nucleotide or amino acid substitutions per site. HAdV-3-TZ01 is highlighted in red. Detailed information on the reference strains used for phylogenetic analysis is provided in Supplementary Table S1.

To further characterize the antigenic features of the isolate, the seven hypervariable regions (HVR1–HVR7) within the hexon gene were analyzed and compared with previously reported HAdV-3 variants (Duan et al., 2021). Sequence alignment revealed that HAdV-3-TZ01 exhibited complete sequence identity across all seven HVRs with the globally circulating HAdV-3 variant 3Hv-2 (Supplementary Figure S1). In contrast, several nucleotide differences were observed when compared with the prototype GB strain, particularly within HVR1, HVR6, and HVR7.

The hexon HVRs constitute the major neutralization epitopes of adenoviruses and are closely associated with antigenic diversity (Haque et al., 2018). The observed HVR profile therefore indicates that HAdV-3-TZ01 belongs to the contemporary 3Hv-2 lineage that has been widely detected among circulating HAdV-3 strains. These findings further support the classification of HAdV-3-TZ01 as a contemporary clinical HAdV-3 isolate and provide additional evidence for its genetic relatedness to currently circulating epidemic strains.

Genome-wide recombination analysis identified a short putative HAdV-7-derived fragment within the viral genome (Supplementary Figure S2). Similar recombination patterns have been reported previously among circulating species B adenoviruses and therefore were not investigated further in the present study (Duan et al., 2021; Rebelo-de-Andrade et al., 2010).

### 3.2 Comparative assessment of apoptosis induction by HAdV-3-TZ01 and HAdV-5 in 293F cells

During virus isolation and propagation, HAdV-3-TZ01 consistently produced visible cytopathic effects (CPE) earlier than laboratory-adapted HAdV-5 under comparable culture conditions. This observation prompted a comparative assessment of apoptosis induction between the two viruses.

To compare the cytopathic characteristics of HAdV-3-TZ01 and HAdV-5 during virus spread, 293F cells were infected at a low multiplicity of infection (MOI = 0.001), and apoptosis was analyzed by Annexin V/PI staining at 48 h post-infection (Figures 3A–C). It should be noted that the two viruses were used at the same nominal MOI based on TCID₅₀ titers; however, as HAdV-3 and HAdV-5 are known to differ in receptor usage, equal TCID₅₀-based MOIs do not necessarily reflect equivalent viral entry efficiency. HAdV-3-TZ01 infection resulted in a statistically significant increase in the proportion of late apoptotic cells (Annexin V^+^/PI^+^, Q2), reaching 14.5%, compared with 8.1% in mock-infected controls and 8.6% in HAdV-5-infected cultures (Figure 3D). Consistently, the total apoptotic population (Q2 + Q3) increased to approximately 19.9% following HAdV-3-TZ01 infection, whereas a smaller increase was observed in the HAdV-5 group (Figure 3E).

**Figure 3:**
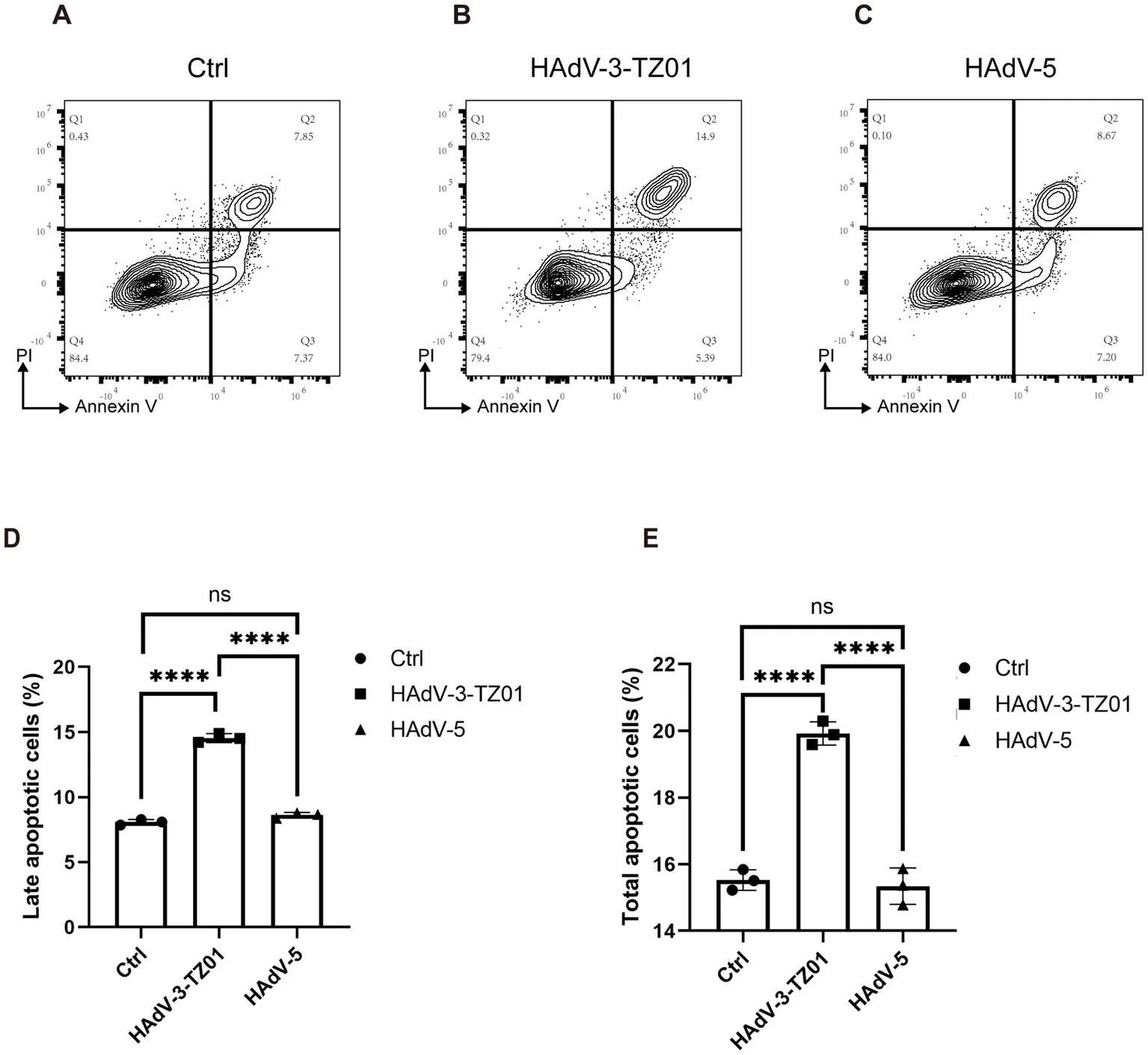
Comparative assessment of apoptosis induction by HAdV-3-TZ01 and HAdV-5 in 293F cells. **(A–C)** Representative Annexin V-FITC/PI flow cytometry contour plots of 293F cells collected 48 h after infection. **(A)** Ctrl (mock-treated cells), **(B)** HAdV-3-TZ01-infected cells, and **(C)** HAdV-5-infected cells. Cell populations were classified as viable cells (Q4), early apoptotic cells (Q3, Annexin V^+^/PI^−^), late apoptotic cells (Q2, Annexin V^+^/PI^+^), and necrotic cells (Q1, Annexin V^−^/PI^+^). **(D)** Quantification of late apoptotic cells (Q2, Annexin V^+^/PI^+^). **(E)** Quantification of total apoptotic cells (Q2 + Q3, Annexin V-positive cells). 293F cells were infected with HAdV-3-TZ01 or HAdV-5 at an MOI of 0.001 and harvested at 48 h post-infection for apoptosis analysis. Data are presented as mean ± SEM from three independent biological replicates. Statistical significance was determined by one-way ANOVA followed by Tukey’s multiple-comparisons test. *p < 0.05; ****p < 0.0001; ns, not significant.

Notably, the marked increase in late apoptotic cells relative to the total apoptotic population suggests that a larger fraction of HAdV-3-TZ01-infected cells had reached the late stage of apoptosis at this time point compared with HAdV-5-infected cells. However, the absolute increase in total apoptotic cells relative to mock infection was modest (approximately 4.5 percentage points). Thus, while the difference between HAdV-3-TZ01 and HAdV-5 reached statistical significance in this experimental setting, its biological relevance—particularly in the context of oncolytic activity in solid tumors—requires cautious interpretation. Importantly, since this experiment was performed exclusively in 293F cells (a suspension-adapted HEK293-derived cell line rather than a tumor cell model), the results cannot be directly extrapolated to lung cancer cell lines such as A549 or H3122. These findings therefore do not establish an inherently superior pro-apoptotic mechanism of HAdV-3, and the molecular pathways underlying the observed difference were not investigated in this study.

### 3.3 Viral genome amplification kinetics and cell tropism of HAdV-3-TZ01 in human and murine cell lines

To evaluate the viral genome amplification kinetics and host-cell tropism of HAdV-3-TZ01, a panel of human respiratory epithelial, tumor-derived, and non-human cell lines, including A549, BEAS-2B, H3122, MRC-5, 293FT, and murine L929 cells, were infected at a low multiplicity of infection (MOI = 0.1) (Figure 4A), and intracellular viral genome levels were quantified at different time points by qPCR targeting the adenoviral hexon gene at 12, 24, 48, 72, and 96 h post-infection (hpi) (Figure 4B).

**Figure 4:**
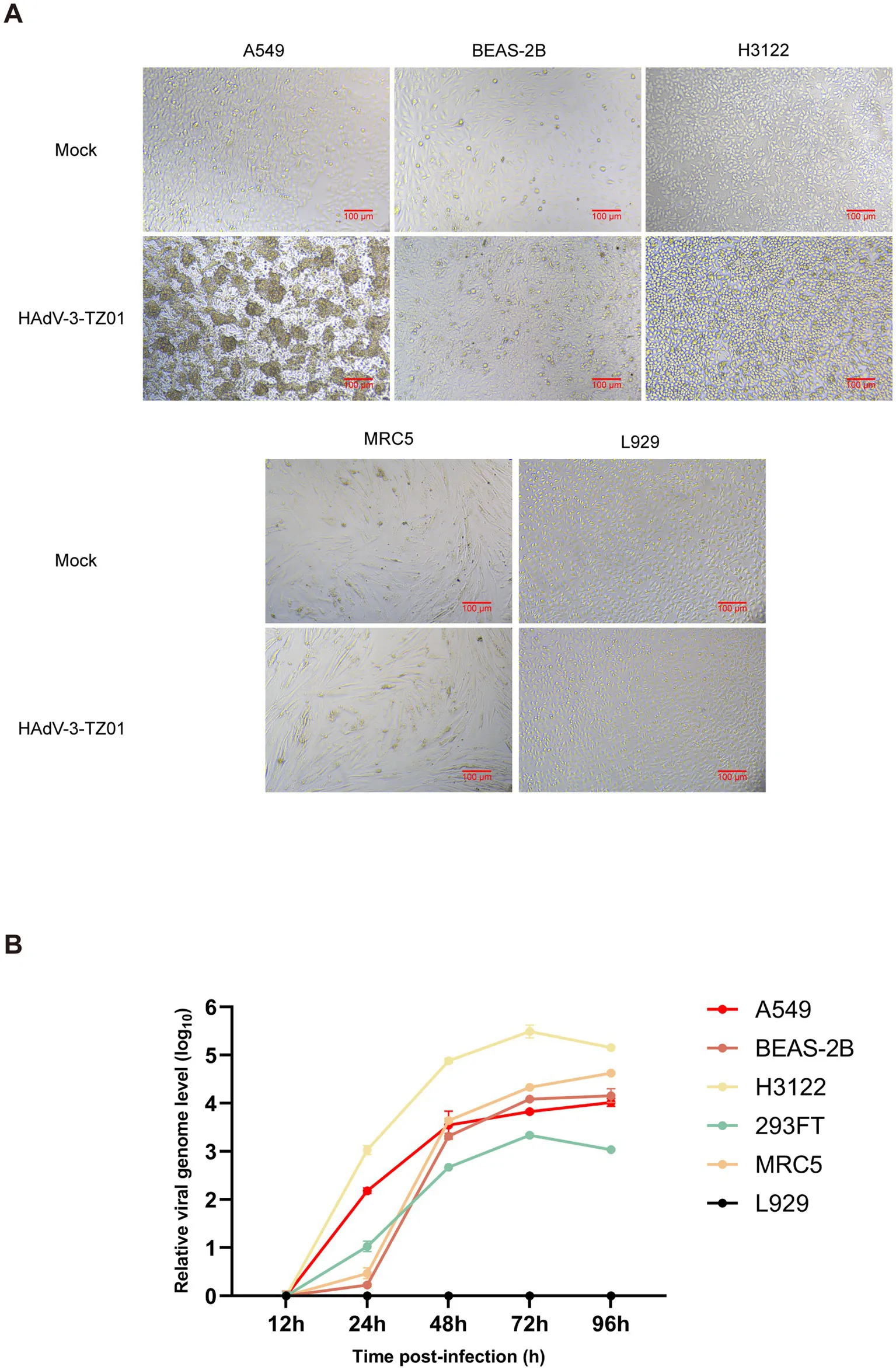
Viral genome amplification and cytopathic effects of HAdV-3-TZ01 in different cell lines. **(A)** Representative cytopathic effects induced by HAdV-3-TZ01 in different cell lines. Representative bright-field micrographs of A549, BEAS-2B, H3122, MRC-5, and L929 cells at 48 h post-infection (hpi) with HAdV-3-TZ01 (MOI = 0.1). Mock-infected cells served as controls. Typical virus-induced cytopathic effects, including cell rounding, detachment, and aggregation, were observed in susceptible human cell lines, whereas no obvious cytopathic changes were detected in murine L929 cells. Scale bar = 100 μm; magnification 100×; imaging conditions (bright-field microscopy). **(B)** Viral genome amplification kinetics of HAdV-3-TZ01 in different cell lines. Human-derived (HEK293FT, BEAS-2B, H3122, MRC-5, and A549) and murine-derived (L929) respiratory-related cell lines were infected with HAdV-3-TZ01 at an MOI of 0.1 based on TCID50 titration. Cells were harvested at 12, 24, 48, 72, and 96 h post infection (hpi), and intracellular adenoviral genome levels were quantified by qPCR targeting the hexon gene. Relative viral genome levels were normalized to the 12 hpi sample within each cell line, which was defined as the baseline level, and presented as log10-transformed values. Mock-infected cells were not used as calibrators because they lack adenoviral hexon templates, making *Ct*_hexon values undetectable and Δ*Ct* incalculable. Data are presented as mean ± SEM from three independent biological experiments (*n* = 3). Statistical analysis was performed using two-way ANOVA followed by Šídák’s multiple comparisons test. Significant differences are indicated as *p < 0.05, **p < 0.01, ***p < 0.001, and ****p < 0.0001; s, not significant.

As shown in Figure 4B, HAdV-3-TZ01 exhibited distinct genome accumulation patterns among the tested cell lines. In human-derived cell lines, including HEK293FT, BEAS-2B, H3122, MRC-5, and A549 cells, viral genome levels progressively increased after infection. Compared with the 12 hpi baseline, significant increases in viral genome levels were observed from 24 hpi onward in most human cell lines. Among these cells, H3122 cells showed the most pronounced viral genome accumulation, with approximately 10^5^-fold increase by 72 hpi compared with the 12 hpi baseline. A549 cells also supported efficient viral genome accumulation, showing a continuous increase throughout the observation period.

In contrast, L929 cells, a murine respiratory-related cell line, showed no significant increase in viral genome levels during the 96 h observation period, suggesting limited genome amplification of HAdV-3-TZ01 in this cell background.

Two-way ANOVA revealed significant effects of cell type, infection time, and their interaction on viral genome accumulation (cell type: *p* < 0.0001; time: *p* < 0.0001; interaction: *p* < 0.0001). Šídák’s multiple comparisons further confirmed significant increases in viral genome levels between 12 hpi and later time points in HEK293FT, BEAS-2B, H3122, MRC-5, and A549 cells, whereas no significant changes were detected in L929 cells. Detailed pairwise comparisons are provided in Supplementary Table S2.

Together, these results demonstrate that HAdV-3-TZ01 efficiently accumulates viral genomes in multiple human respiratory-related cell types, including both non-transformed and tumor-derived cells, while showing limited replication capacity in murine cells.

### 3.4 HAdV-3-TZ01 suppresses tumor growth *in vivo*

#### 3.4.1 HAdV-3-TZ01 suppresses tumor growth in A549 xenografts

To evaluate the antitumor activity of HAdV-3-TZ01 *in vivo*, an A549-luciferase xenograft model was established in BALB/c-nu nude mice. When tumors reached approximately 100 mm^3^, mice were randomly assigned to receive intratumoral administration of HAdV-3-TZ01 (50 μL per dose, 3.16 × 10^11^ TCID₅₀/mL) or PBS as a mock treatment control (Figure 5A).

**Figure 5:**
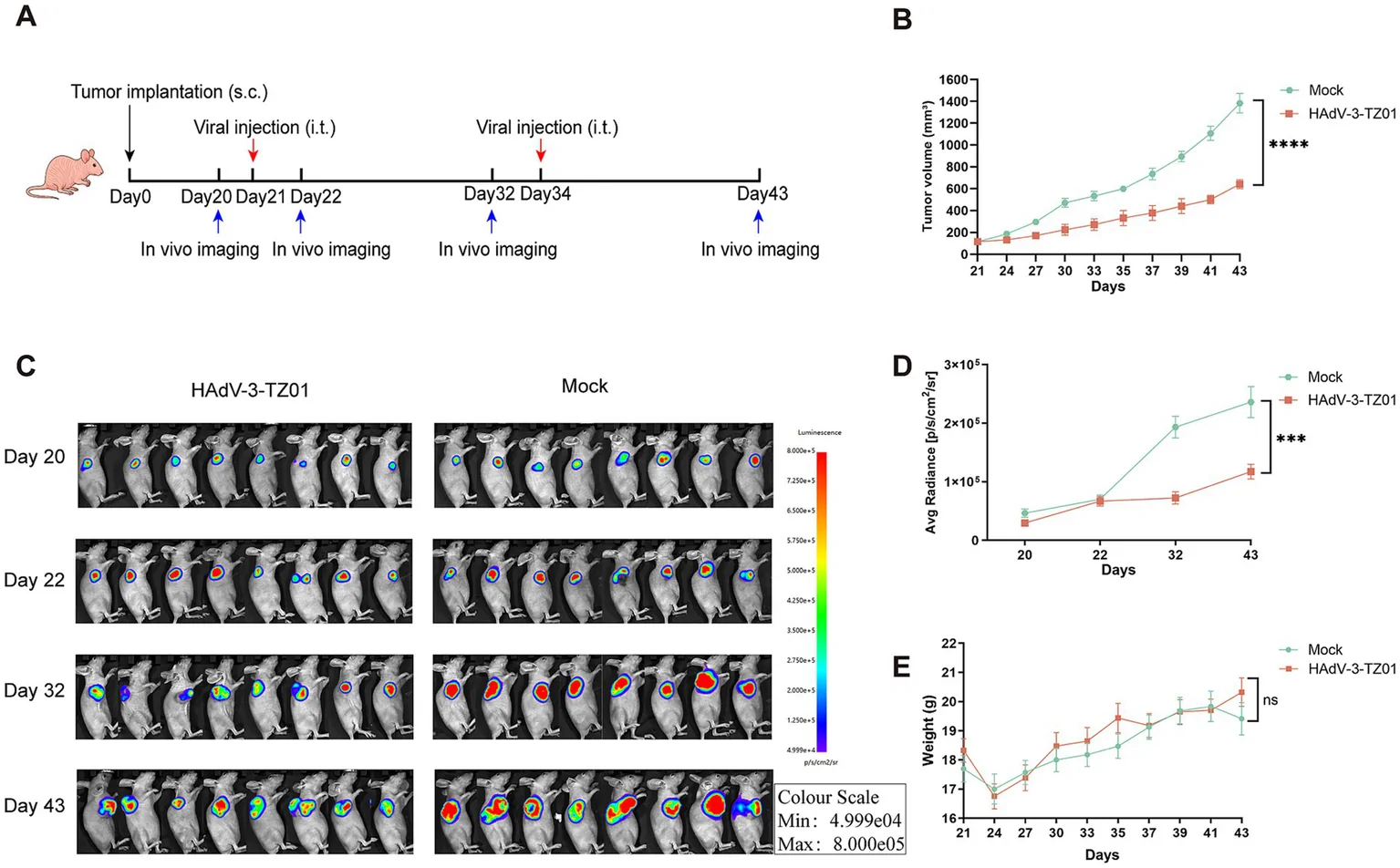
Antitumor activity of HAdV-3-TZ01 in an A549 xenograft model. **(A)** Schematic overview of the *in vivo* experimental design. BALB/c-nu mice bearing subcutaneous A549-LUC xenograft tumors were treated with intratumoral injections of HAdV-3-TZ01 or PBS at the indicated time points. Tumor growth and treatment response were monitored by serial bioluminescence imaging (BLI) during the study period. **(B)** Tumor growth curves of mice treated with HAdV-3-TZ01 or PBS. Tumor volume was calculated as *V* = (*L* × *W*^2^)/2, where *L* represents the longest diameter and *W* represents the shortest perpendicular diameter. **(C)** Representative bioluminescence images of mice from the HAdV-3-TZ01-treated group and PBS control group at the indicated time points. **(D)** Quantitative analysis of tumor-associated bioluminescence signal intensity. Data are presented as mean ± SEM (*n* = 8 per group). **(E)** Body weight changes of tumor-bearing mice throughout the study period. Data are presented as mean ± SEM (*n* = 8 per group). For panels **(B,D,E)**, statistical significance was determined by two-way repeated-measures ANOVA with Geisser–Greenhouse correction followed by multiple-comparisons tests. *p < 0.05, **p < 0.01, ***p < 0.001, ****p < 0.0001.

At the initiation of treatment, tumor volumes were comparable between the two groups. In the mock-treated group, tumors exhibited continuous and rapid growth throughout the observation period, reaching a mean volume of 1383.5 mm^3^ on day 43. In contrast, HAdV-3-TZ01 treatment markedly delayed tumor progression. Differences in tumor growth became evident from day 24 and progressively increased during subsequent monitoring. By the end of the experiment, the mean tumor volume in the HAdV-3-TZ01-treated group was reduced to 642.2 mm^3^, representing an approximately 54% decrease compared with the mock-treated group (Figure 5B).

Throughout the study, tumors receiving HAdV-3-TZ01 displayed substantially slower growth kinetics, whereas tumors in the mock-treated group showed accelerated progression. These results demonstrate that intratumoral administration of HAdV-3-TZ01 effectively suppresses tumor growth in an A549 xenograft model, supporting its potential as an oncolytic adenovirus candidate. However, whether the observed antitumor effect is primarily attributable to sustained viral replication and intratumoral spread warrants further investigation.

#### 3.4.2 Bioluminescence imaging confirms reduced tumor burden

To further evaluate the antitumor efficacy of HAdV-3-TZ01 *in vivo*, longitudinal bioluminescence imaging (BLI) was performed on tumor-bearing mice at days 20, 22, 32, and 43 following tumor implantation (Figure 5C). Prior to treatment initiation, bioluminescence signals were comparable between the two groups, indicating similar baseline tumor burdens.

During the early stage of treatment (day 22), luminescence intensities remained similar between virus-treated and mock-treated animals, suggesting that viral replication and oncolytic activity had not yet produced measurable differences in tumor burden. However, clear divergence emerged at later time points. By day 32, the mean bioluminescence signal in the mock-treated group had increased to approximately 1.93 × 10^5^ photons/s/cm^2^/sr, whereas the corresponding signal in the HAdV-3-TZ01-treated group remained substantially lower at approximately 7.54 × 10^4^ photons/s/cm^2^/sr.

This difference persisted through the end of the observation period. At day 43, the mean luminescence intensity in the mock group reached approximately 2.37 × 10^5^ photons/s/cm^2^/sr, compared with 1.17 × 10^5^ photons/s/cm^2^/sr in virus-treated animals, representing an approximately 50% reduction in tumor-associated bioluminescence signal. Representative imaging further demonstrated markedly weaker luminescence in the HAdV-3-TZ01-treated mice than in control animals.

Longitudinal bioluminescence imaging further confirmed suppression of tumor progression, as luminescence signals remained substantially lower in virus-treated animals than in mock-treated controls (Figure 5D). Statistical analysis demonstrated a significant treatment effect over time. To assess the tolerability of HAdV-3-TZ01 treatment, body weights were monitored throughout the study (Figure 5E). Both virus-treated and mock-treated mice exhibited a gradual increase in body weight over the observation period. Although a transient decrease was observed in several animals shortly after the first intratumoral injection, body weights recovered rapidly and continued to increase thereafter. No progressive weight loss was detected following either the first (day 21) or second (day 34) viral administration. Furthermore, no overt signs of distress, impaired mobility, or treatment-related mortality were observed. These findings indicate that, under the specific conditions of this xenograft study, intratumoral administration of HAdV-3-TZ01 was tolerated, with no significant body weight loss or overt signs of distress observed. However, comprehensive safety evaluation—including histopathological examination of major organs and assessment of viral dissemination—was not performed in this study.

Together, these findings demonstrate that intratumoral administration of HAdV-3-TZ01 significantly suppressed tumor progression in the A549 xenograft model and was well tolerated under the xenograft conditions, with no detectable toxicity observed.

## 4 Discussion

In the present study, we isolated and characterized a contemporary clinical HAdV-3 strain, HAdV-3-TZ01. Genomic analysis confirmed that HAdV-3-TZ01 belonged to HAdV-3 and retained the characteristic genetic features of currently circulating strains. Functional characterization revealed robust viral genome replication in human respiratory epithelial cells and multiple epithelial-derived tumor cell lines. Intratumoral administration significantly inhibited tumor growth in an A549 xenograft model, with no detectable body weight loss or overt signs of distress under the experimental conditions. These findings provide updated biological insights into a naturally circulating HAdV-3 isolate and suggest its potential value as an alternative adenoviral backbone for oncolytic virotherapy.

The viral genome replication characteristics of HAdV-3-TZ01 were consistent with the known epithelial tropism of species B adenoviruses. Viral genome levels increased rapidly in H3122, BEAS-2B, and A549 cells, whereas replication was substantially restricted in murine L929 cells, consistent with the species specificity of human adenoviruses. Importantly, HAdV-3-TZ01 also showed active viral genome replication in several human tumor cell lines under low multiplicity conditions, suggesting that malignant epithelial cells provide a suitable environment for viral amplification. However, we did not assess receptor expression levels (CD46, DSG2, or CAR) in the cell lines tested, nor did we directly compare viral replication or cytotoxicity between malignant and non-malignant human cells. Such comparisons will be important for evaluating tumor selectivity in future studies.

HAdV-3-TZ01 infection induced apoptotic cell death in infected cells. However, the apoptosis analysis in the present study was performed exclusively in 293F cells (a non-tumorigenic HEK293-derived cell line), and the absolute increase in total apoptotic cells relative to mock infection was modest (approximately 4.5 percentage points). Moreover, equal TCID₅₀-based MOIs do not guarantee equivalent viral entry efficiency due to distinct receptor usage, and we did not assess early infection kinetics or apoptosis-associated molecular markers. Whether HAdV-3-TZ01 induces enhanced apoptosis in tumor-relevant cell lines such as A549 or H3122, or exhibits differential cytotoxicity compared with non-malignant epithelial cells such as BEAS-2B, remains to be determined. Previous studies have demonstrated that HAdV-3 infection can activate an intrinsic apoptosis-associated cell death program in A549 cells, accompanied by the release of extracellular vesicles containing cellular stress and apoptotic markers. These findings provide mechanistic support for further investigation of apoptosis-related pathways induced by HAdV-3-TZ01, particularly in tumor-relevant cellular contexts. Most clinically evaluated oncolytic adenoviruses have been developed based on engineered HAdV-5 platforms. Species B adenoviruses, including HAdV-3, utilize distinct cellular receptors from HAdV-5 and may therefore exhibit different patterns of cellular tropism. Previous studies have demonstrated the feasibility of HAdV-3 as an oncolytic platform; for example, the engineered HAdV-3-based vector Ad3-hTERT-E1A showed selective replication and antitumor activity across multiple cancer types while maintaining reduced toxicity in non-malignant cells (Hemminki et al., 2011). However, these studies primarily focused on genetically modified viral vectors optimized for therapeutic applications. In contrast, HAdV-3-TZ01 represents a naturally occurring clinical isolate that showed antitumor activity *in vitro* and *in vivo* without prior genetic modification. Therefore, the novelty of this study does not lie in demonstrating that HAdV-3 can infect tumor cells, but rather in providing a comprehensive characterization of a contemporary circulating HAdV-3 isolate by integrating genomic features, comparative replication properties, and preliminary *in vivo* antitumor activity. HAdV-3-TZ01 is a wild-type respiratory pathogen that retains efficient viral genome amplification in non-malignant bronchial epithelial cells (BEAS-2B); therefore, rather than serving as an immediate therapeutic agent, this isolate provides a valuable starting platform for future genetic engineering aimed at achieving tumor selectivity and attenuated pathogenicity. Genomic analysis identified a short HAdV-7-derived recombinant fragment within the HAdV-3-TZ01 genome. Despite this recombinant region, the overall genomic architecture and major structural proteins remained highly conserved. Whether this fragment contributes to the biological characteristics of HAdV-3-TZ01 requires further investigation.

The antitumor effect observed in the A549 xenograft model—approximately 54% reduction in tumor volume and ~50% reduction in bioluminescence signal at day 43—provides consistent evidence of antitumor activity following intratumoral administration. The A549 xenograft model has been widely used for evaluating the *in vivo* efficacy of oncolytic adenoviruses, including conditionally replicative adenovirus platforms, allowing comparison with previous reports. For instance, studies on the well-known oncolytic adenovirus ONYX-015 have employed this model to demonstrate its antitumor efficacy following systemic or intratumoral administration (Heise et al., 1999). More recently, the A549 xenograft model has also been used to evaluate armed conditionally replicating adenoviruses, such as a p14 FAST protein-expressing vector, which induced widespread syncytium formation and reduced tumor growth rates *in vivo* (Del Papa et al., 2019). Although H3122 cells showed the highest viral genome accumulation *in vitro*, A549-Luc cells were selected for the *in vivo* studies because they provide consistent tumor establishment, reproducible growth kinetics, and reliable longitudinal bioluminescence monitoring. Nevertheless, direct evidence linking the observed tumor inhibition to sustained viral replication and intratumoral spread remains unavailable. Specifically, we did not evaluate longitudinal viral genome kinetics or recover infectious progeny viruses from tumor tissues, nor did we perform spatial analyses of viral distribution through adenoviral antigen staining or functional assessment of tumor cell death by histological markers. These limitations do not negate the observed antitumor efficacy but highlight the need for future studies integrating virological and pathological analyses to define the mechanisms underlying HAdV-3-TZ01-mediated tumor suppression. The dosing schedule employed in this study (two intratumoral injections on days 21 and 34, with a 13-day interval) is supported by precedents in the field, where repeated intratumoral administrations at approximately 2-week intervals have been evaluated in both preclinical and clinical settings. For instance, in the phase 1 BETA prime trial, the oncolytic adenovirus AdAPT-001 was administered intratumorally once every 2 weeks at the recommended phase 2 dose (Conley et al., 2024). Furthermore, studies have suggested that increasing the number of injections, rather than the dose per injection, may be a more effective strategy to enhance therapeutic outcomes.

Several limitations of the present study should be acknowledged. First, viral replication was evaluated primarily through intracellular viral DNA quantification, which does not directly measure infectious progeny production. Second, the apoptosis analysis was limited to 293F cells and did not include tumor-relevant or non-malignant epithelial controls. Third, receptor expression levels were not examined, and early entry efficiency was not assessed. Fourth, *in vivo* evaluation was limited to a single xenograft model with intratumoral administration, constraining generalizability rather than undermining the A549 results. Fifth, because experiments were conducted in immunodeficient mice, the contribution of host immune responses could not be assessed. Sixth, we did not perform histopathological examination of major organs to assess systemic toxicity. Future studies incorporating additional tumor models, mechanistic analyses, and immunocompetent systems will be necessary to fully evaluate the therapeutic potential of HAdV-3-TZ01.

In summary, we isolated and characterized a contemporary clinical HAdV-3 isolate with antitumor activity in a xenograft model. HAdV-3-TZ01 demonstrated efficient genome replication in human epithelial-derived cells, induced tumor cell death, and suppressed tumor growth *in vivo*. As an unmodified respiratory pathogen, it represents a potential starting backbone for future engineering efforts aimed at enhancing tumor selectivity and safety, rather than a directly translatable therapeutic agent.

## Data Availability

The complete genome sequence of HAdV-3-TZ01 generated in this study has been deposited in GenBank under accession number PZ163190. All other data supporting the findings of this study are available from the corresponding author upon reasonable request.
