# Supplementary material for: Biological characterization of a clinical human adenovirus type 3 isolate with oncolytic potential
Source: Front Microbiol. 2026 Sep 23;17:1912909. doi: 10.3389/fmicb.2026.1912909 (PMC13642665; doi:10.3389/fmicb.2026.1912909)
Supplement: Supplementary file 1 [file Supplementary_file_1.DOCX]

Supplementary Material

# 1 Supplementary Figures and Tables

## 1.1 Supplementary Table

| Adenovirus | Country/Place of Origin | | Year of Collection | Strain | Year of Sequence Submission | GenBank Accession No |
| --- | --- | --- | --- | --- | --- | --- |
| HAdV-2 | — | — | | — | 1996 | J01917.1 |
| HAdV-3 | USA | 1953 | | GB | 2004 | AY599834 |
| HAdV-4 | USA | 1953 | | RI-67 | 2016 | KX384949 |
| HAdV-5 | Sweden | 2000 | | GyK193 | 2016 | KX868466.2 |
| HAdV-7 | USA | ­­­— | | Gomen | 2004 | AY594255.1 |
| HAdV-9 | Germany | — | | Hicks | 2004 | AJ854486.1 |
| HAdV-11 | Sweden | — | | Slobitski | 2002 | AF532578 |
| HAdV-12 | Sweden | 1978 | | GyK010 | 2016 | KX868289.2 |
| HAdV-16 | USA | — | | ch.79 | 2004 | AY601636 |
| HAdV-26 | The Netherlands | — | | — | 2006 | EF153474.1 |
| HAdV-41 | China | 2021 | | GZ78 | 2022 | OP019370.1 |
| HAdV-52 | USA | — | | T03-2244 | 2005 | DQ923122.2 |

**Supplementary Table S1.** Human adenovirus strains used for phylogenetic analysis.

Information on the human adenovirus strains included in the phylogenetic analyses, including adenovirus type, country or region of origin, year of isolation, strain designation, year of sequence submission to GenBank, and GenBank accession number. The dataset comprises representative strains of HAdV-2, HAdV-3, HAdV-4, HAdV-5, HAdV-7, HAdV-9, HAdV-11, HAdV-12, HAdV-16, HAdV-26, HAdV-41, and HAdV-52. A dash (—) indicates that the corresponding information was unavailable.

## A

| **Source of variation** | **F value** | **Degrees of freedom** | **P value** | **Significance** |
| --- | --- | --- | --- | --- |
| Cell type | F(5,60) = 970.5 | 5,60 | <0.0001 | **** |
| Infection time | F(4,60) = 1934 | 4,60 | <0.0001 | **** |
| Cell type × Infection time interaction | F(20,60) = 111.1 | 20,60 | <0.0001 | **** |

## B

| **Cell line** | **Comparison** | **Adjusted P value** | **Significance** |
| --- | --- | --- | --- |
| **L929** | 12 hpi vs 24 hpi | >0.9999 | ns |
|  | 12 hpi vs 48 hpi | >0.9999 | ns |
|  | 12 hpi vs 72 hpi | >0.9999 | ns |
|  | 12 hpi vs 96 hpi | >0.9999 | ns |
| **HEK293FT** | 12 hpi vs 24 hpi | <0.0001 | **** |
|  | 12 hpi vs 48 hpi | <0.0001 | **** |
|  | 12 hpi vs 72 hpi | <0.0001 | **** |
|  | 12 hpi vs 96 hpi | <0.0001 | **** |
| **BEAS-2B** | 12 hpi vs 24 hpi | 0.1276 | ns |
|  | 12 hpi vs 48 hpi | <0.0001 | **** |
|  | 12 hpi vs 72 hpi | <0.0001 | **** |
|  | 12 hpi vs 96 hpi | <0.0001 | **** |
| **H3122** | 12 hpi vs 24 hpi | <0.0001 | **** |
|  | 12 hpi vs 48 hpi | <0.0001 | **** |
|  | 12 hpi vs 72 hpi | <0.0001 | **** |
|  | 12 hpi vs 96 hpi | <0.0001 | **** |
| **MRC-5** | 12 hpi vs 24 hpi | 0.0015 | ** |
|  | 12 hpi vs 48 hpi | <0.0001 | **** |
|  | 12 hpi vs 72 hpi | <0.0001 | **** |
|  | 12 hpi vs 96 hpi | <0.0001 | **** |
| **A549** | 12 hpi vs 24 hpi | <0.0001 | **** |
|  | 12 hpi vs 48 hpi | <0.0001 | **** |
|  | 12 hpi vs 72 hpi | <0.0001 | **** |
|  | 12 hpi vs 96 hpi | <0.0001 | **** |

**Supplementary Table S2**. Statistical analysis of HAdV-3-TZ01 genome accumulation in different respiratory-related cell lines.

(A) Two-way analysis of variance (ANOVA) of HAdV-3-TZ01 genome accumulation. The effects of cell type, infection time, and their interaction on viral genome accumulation were analyzed using two-way ANOVA. (B) Šídák’s multiple comparisons test. Pairwise comparisons of HAdV-3-TZ01 genome accumulation between different infection time points within each cell line were performed following two-way ANOVA. Statistical significance was indicated as follows: ns, not significant; P < 0.01 (**); P < 0.0001 (****).

## 1.2 Supplementary Figures

**Supplementary Figure S1.** Sequence alignment analysis of the hypervariable regions (HVR1–HVR7) within the hexon gene of human adenovirus type 3.

A schematic diagram indicates the locations of the seven hypervariable regions (HVR1–HVR7) within the hexon gene. Multiple sequence alignment was performed among HAdV-3-TZ01, the reference strain GB, Chinese epidemic variants (3Hv-2 and 3Hv-14 to 3Hv-20), and globally prevalent variants (3Hv-1 to 3Hv-4). HAdV-3-TZ01 exhibited an HVR pattern identical to that of variant 3Hv-2 and is highlighted in red together with 3Hv-2.

**Supplementary Figure S2.** Recombination analysis of HAdV-3-TZ01.

**(A)** Whole-genome recombination analysis of HAdV-3-TZ01 with representative species B human adenoviruses. Similarity plot and Bootscan analyses were performed using SimPlot with a window size of 500 bp, step size of 200 bp, Kimura two-parameter distance model, and neighbor-joining tree model with 1,000 bootstrap replicates. The GapStrip option was enabled, and the similarity threshold (T/t) was set to 2.0. The Y-axis represents sequence similarity (%) or bootstrap support (%), and the X-axis indicates genome position (bp). Different colored curves represent comparisons with reference strains of species B adenoviruses. Red boxes indicate putative recombination regions. The genomic positions of the protein IIIa precursor (nt 12,053–13,819), penton base (nt 13,907–15,544), protein VII precursor (nt 15,557–16,135), and L2 minor core protein (nt 16,178–17,230) are indicated.

**(B)** Maximum-likelihood phylogenetic tree constructed from complete genome sequences of representative HAdV-3 and HAdV-7 strains, showing the overall genomic relationship of HAdV-3-TZ01 to reference adenoviruses.

**(C)** Maximum-likelihood phylogenetic tree based on the genomic region identified by SimPlot analysis (nt 12,500–16,300), showing the clustering of HAdV-3-TZ01 with the HAdV-7 reference strain Gomen.

**(D)** Maximum-likelihood phylogenetic tree generated from the recombinant fragment identified by RDP4 (nt 12,786–14,744), demonstrating that HAdV-3-TZ01 clusters within the HAdV-7 lineage.
